# A DNA Methylation Network Interaction Measure, and Detection of Network Oncomarkers

**DOI:** 10.1371/journal.pone.0084573

**Published:** 2014-01-06

**Authors:** Thomas E. Bartlett, Sofia C. Olhede, Alexey Zaikin

**Affiliations:** 1 Department of Mathematics, University College London, London, United Kingdom; 2 Department of Statistical Science, University College London, London, United Kingdom; 3 CoMPLEX, University College London, London, United Kingdom; Innsbruck Medical University, Austria

## Abstract

Epigenetic processes–including DNA methylation–are increasingly seen as having a fundamental role in chronic diseases like cancer. DNA methylation patterns offer a route to develop prognostic measures based directly on DNA measurements, rather than less-stable RNA measurements. A novel DNA methylation-based measure of the co-ordinated interactive behaviour of genes is developed, in a network context. It is shown that this measure reflects well the co-regulatory behaviour linked to gene expression (at the mRNA level) over the same network interactions. This measure, defined for pairs of genes in a single patient/sample, associates with overall survival outcome independent of known prognostic clinical features, in several independent data sets relating to different cancer types. In total, more than half a billion CpGs in over 1600 samples, taken from nine different cancer entities, are analysed. It is found that groups of gene-pair interactions which associate significantly with survival identify statistically significant subnetwork modules. Many of these subnetwork modules are shown to be biologically relevant by strong correlation with pre-defined gene sets, such as immune function, wound healing, mitochondrial function and MAP-kinase signalling. In particular, the wound healing module corresponds to an increase in co-ordinated interactive behaviour between genes for worse prognosis, and the immune module corresponds to a decrease in co-ordinated interactive behaviour between genes for worse prognosis. This measure has great potential for defining DNA-based cancer biomarkers. Such biomarkers could naturally be developed further, by drawing on the rapidly expanding knowledge base of network science.

## Introduction

Epigenetic information is stored in the genome in the form of heritable modifications to the chemical structure of DNA, such as methylation of CpG di-nucleotides. Epigenetic information can be modulated during the lifetime of an organism by environmental cues [1]–[3] and these changes persist in subsequent mitoses, leading to an acquired change of phenotype. As such, epigenetics can be considered to be an interface between the genome and the environment, and consequently also a conduit for environmental risk factors.

Alterations in DNA methylation (DNAm) levels are among the earliest changes in human carcinogenesis [1], and hence offer novel strategies to identify individuals who might be at risk of developing such illnesses or individuals with early stage cancers. However, to proceed with developing such tests, measures relating to DNA methylation are needed, which can be consistently linked to clinically relevant differences, such as patient outcome. Per-gene measures of DNA methylation have been shown to be relevant to the study of cancer genomics [4], and network models and measures naturally reflect the collective behaviour of groups of similar items, such as genes, and their interactions with one another. As such, they may help with the development of DNAm-based biomarkers which take account of such collective and interactive behaviour of genes.

As a cancer progresses, its signalling and control networks are re-arranged (‘re-wired’), and this drives adaptive alterations in phenotype, which are advantageous for the cancer [5]. Previous research by other authors [6] found that patient survival outcome in breast cancer (BRCA) could be predicted well by network models of this re-wiring, based on gene expression data. DNAm patterns are more stable than gene expression patterns, because DNAm measurements are taken directly from DNA, whereas gene expression measurements must come via RNA. Hence, DNAm patterns might be expected to lead to more reliable disease biomarkers than gene expression patterns would be able to. Here, we develop a measure of network interaction ‘co-ordinatedness’, between pairs of genes, based on DNA methylation data. We show that this measure, calculated for pairs of genes, is highly associated with co-regulatory behaviour linked to gene expression (at the mRNA level) over the same network interactions/pairs of genes. We show that this measure associates significantly with overall survival outcome independent of known prognostic markers in several data sets and cancer types, and that groups of these significant gene-gene network interactions identify subnetwork modules, with a well-controlled false discovery rate. Of these significant subnetwork modules, one module corresponds very strongly to wound healing, with its gene-gene interactions displaying an increase in co-ordinated behaviour with worse disease prognosis. Another of these significant subnetwork modules corresponds very strongly to immune function, with its gene-gene interactions predominantly showing a decrease in co-ordinated behaviour with worse disease prognosis. Our findings provide the basis for further development of independent DNA methylation oncomarkers in the context of network science.

## Results

### The DNA Methylation Network Correlation Measure

The DNA methylation (DNAm) network correlation measure quantifies the extent to which the DNA methylation profiles of a pair of genes ‘explain’ each other. It is based only on measurements of the DNA methylation profiles of this pair of genes, and it acts as a surrogate for a measure of the extent to which this pair of genes behave interactively. Such interactive behaviour may include transcriptional regulation or other types of biochemical interaction, and a pair of genes will affect each other’s transcriptional behaviour or other biochemical functioning most directly as a result of their own expression levels. An increase in methylation level around the gene promoter is certainly correlated with a decrease in the expression level of the gene, although which of these occurs first is not clear [7]. An increase in methylation level in the gene body is related less certainly to effects on gene transcriptional and translational behaviour, which may include increased expression level and alternatively spliced gene products [7]. Hence, there may be a number of components of the variation of the methylation profile of a gene which are significant in terms of their correlation with different transcriptional effects of that gene. The methylation profile of a gene is taken as a surrogate for these various interactive effects in the DNAm network cross correlation measure, which quantifies the extent to which these interactive effects or patterns in a pair of genes explain each other, as reflected in the DNA methylation profiles.

The DNAm network cross correlation measure is defined by analogy to Canonical Correlation Analysis (CCA) [8] (see ‘methods and models’ for formal definitions). CCA aims to discover linear combinations of variables of one type, and linear combinations of variables of another type, so that these combinations best ‘explain’ each other. In this context, a particular way of combining (by scaling and adding) the deviations from the mean methylation profile at a number of locations within one gene might be particularly effective at explaining a particular combination (again, by scaling and adding) of the deviations from the mean methylation profile at a number of locations in another gene, and *vice-versa*. There will probably be fewer ways in which the methylation levels of these genes vary across the samples, than there are locations at which methylation is measured, along each gene; this is because the methylation level at many locations along a gene is highly correlated. CCA finds the most important components of this variation across samples (in terms of these linear combinations of variables of each type, i.e., methylation measurements in each gene) which both these types of variables (i.e., DNA methylation in the two genes considered) have in common.

**Figure 1:**
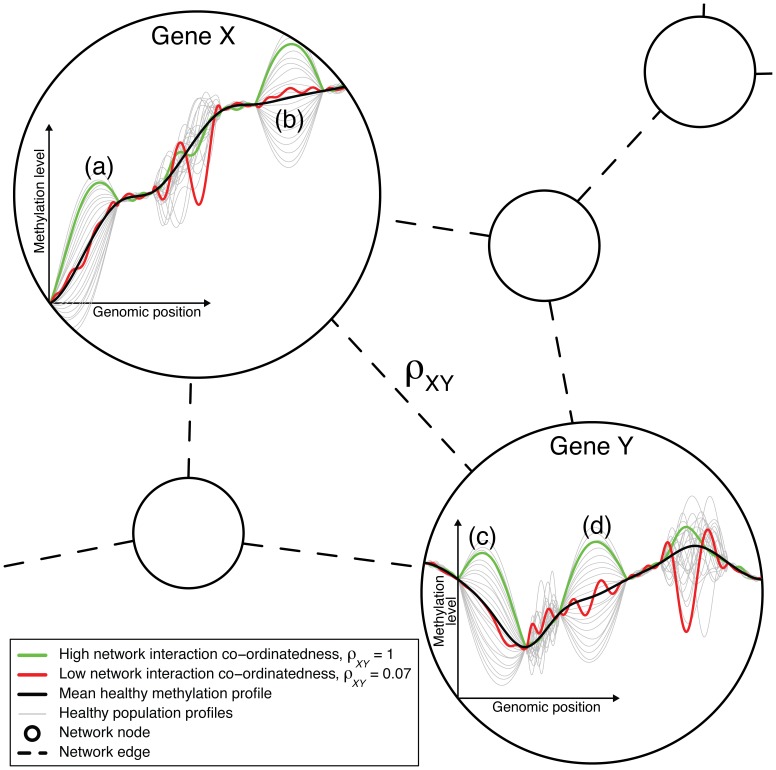
The DNA methylation network interaction measure. A combination of the variation of the healthy methylation profiles in regions (a) and (b) of gene X explains well/is well-explained by a combination of the variation of the healthy methylation profiles in regions (c) and (d) of gene Y. The green cancer sample varies by a large amount about the mean methylation profile and in a typical way in these regions in both genes. Hence, the green sample corresponds to a high level of network interaction co-ordinatedness, as measured by the DNA methylation network interaction measure, 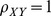. The variation in the other regions of these genes do not well-explain each other, and so the red sample, which varies by a large amount in these other regions and varies less and in an atypical way in regions (a)–(d), corresponds to a low level of network interaction co-ordinatedness, 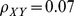. Genes X and Y are likely to have different numbers of methylation measurement locations (i.e., variables X and Y are of different dimension). The ordering of the measurement locations has no influence on the calculation of 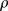, as long as the ordering is consistent across samples.

CCA finds linear combinations of the two types of variables that covary. Because linear combinations are evaluated, the two types of variables can be of a different number. The DNAm network cross correlation measure therefore evaluates the extent to which, in an individual sample, these combinations in this pair of types of variables explain each other. The covariation is assessed against typical variability in such variables. Such variation is assessed in terms of the evaluated statistics. Variation is understood in terms of the population covariance matrix, inherent to healthy samples, the methylation profile for one gene makes up the variables of one type, and the variables of that type are the methylation points, i.e., CpGs, along that gene. There are many sources of variation. When the DNAm network cross correlation measure is large (i.e., close to 1), the corresponding pair of genes explain each others transcriptional or translational behaviour (as reflected in their methylation profiles) well, or have otherwise well-correlated interactive behaviour, for the corresponding sample (patient); see figure 1.

### Application of the DNA Methylation Network Correlation Measure to Data

The DNAm network correlation measure was developed and evaluated in the context of DNAm data obtained via the Illumina Infinium Human Methylation 450 K platform, from samples from cancer patients made publicly available via The Cancer Genome Atlas (TCGA) project [9]–[11], relating to nine different cancer types. Details of the number of samples (patients) for which DNA methylation and survival analysis were carried out, for each data set, appear in table 1.

**Table 1:** Number of samples in each data set.

	Samples	Events
BLCA	108	31
BRCA	173	23
COAD	237	36
HNSC	266	89
KIRC	265	91
LIHC	50	20
LUAD	143	45
LUSC	141	48
UCEC	276	28

Number of samples (patients) in each data set, together with number of events (i.e., number of patients who died before the end of the respective study). Abbreviations as follows: Bladder Urothelial Carcinoma (BLCA), Breast Invasive Carcinoma (BRCA), Colon Adenocarcinoma (COAD), Head and Neck Squamous Cell Carcinoma (HNSC), Kidney Renal Clear Cell Carcinoma (KIRC), Kidney Renal Papillary Cell Carcinoma (KIRP), Liver (LIHC), Lung Adenocarcinoma (LUAD), Lung Squamous Cell Carcinoma (LUSC), and Uterine Corpus Endometrioid Carcinoma (UCEC).

For each data set, considering all possible pairs of the 14800 genes available would require more than 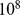 comparisons, each based on the data for a pair of genes across all the samples in that data set. Further, it is possible that spurious correlations could arise if all possible pairs of genes were considered in this way. For example, the expression and regulatory patterns of a pair of genes may be highly correlated if they are both part of the same signalling pathway, even if they do not directly interact. To avoid problems due to the high number of tests inherent to considering all pairwise gene interactions, we restricted the number of pairs of genes for which the DNAm network correlation measure was considered. The DNAm network correlation measure was only calculated for a pair of genes, and subsequent analyses were only carried out, if that pair of genes was listed as taking part in a known biochemical interaction. We note that [6] proposed such a restriction for pairwise gene interactions, and applied it successfully. A list of such interactions was downloaded from http://www.pathwaycommons.org, and was used as a canonical human interactome map.

### Comparing the DNA Methylation Network Correlation Measure with Gene Expression Network Correlation

In order to test whether a major component of the interactive/co-regulatory behaviour quantified by the DNA methylation network correlation measure corresponds to that which can be measured by mRNA levels, the following procedure was carried out. For the five data sets included in the subsequent DNA methylation analysis for which sufficient gene expression data (in the form of mRNA levels) were available, for each pair of genes for which the DNAm network correlation measure was calculated, the Spearman correlation coefficient of the mRNA expression levels for that pair of genes was calculated, across all the cancer samples with gene expression data available for that tumour type. For each of these pairs of genes, the mean of the DNA methylation network correlation measure was also calculated across all available (cancer) samples. The significance of association of the mean DNAm network correlation measure with the mRNA expression (gene expression) network correlation coefficient across all pairs of genes is shown in table 2, for each data set. Note that table 2 shows different numbers of samples for each cancer type to table 1, because the DNA methylation and gene expression analyses were based mostly on different samples from the same cancer type. Absolute values of these correlation measures are compared, because negative values mean different things in relation to the DNAm network correlation measure, and to the correlation coefficient of the gene expression data. For each data set, a Spearman correlation test comparing these network correlation measures across all pairs of genes is very significant, with 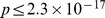 for all five data sets considered. Hence, it is concluded that a major component of the interactive behaviour quantified by the DNAm network correlation measure is the interactive (co-regulatory) behaviour corresponding to gene expression, at the mRNA level.

**Table 2:** Significance of association of the DNAm network correlation measure with gene expression network correlation.

Data set	Samples	*p*-value
BRCA	590	4.4×10^−19^
COAD	174	5×10^−21^
KIRK	72	3.2×10^−17^
LUSC	155	6×10^−38^
UCEC	54	2.3×10^−17^

For each gene-pair for which the DNAm network correlation measure is calculated, for which there are also mRNA expression (gene expression) data available, the Spearman correlation coefficient comparing the expression levels of that pair of genes is calculated across the available samples in a given data set (tumour samples only). For each pair of genes, the mean of the DNAm network correlation measure across the available samples in that data set is also calculated. These mean DNAm network correlation and expression network correlation measures are compared across all pairs of genes for each data set, with the corresponding *p*-values (Spearman correlation test) shown in the table.

In many (approximately one third) of gene-pair comparisons made by the DNAm network correlation measure, it was calculated that there are two or more significant components of covariation between these pairs of genes, as determined by examining the corresponding cross-covariance matrices. Each such calculation was carried out by performing a singular value decomposition to estimate the variances corresponding to the main components of variation, and comparing these to empirical null model variances calculated similarly after randomising the original data, as in previous genomics studies by other authors [12]. The most likely candidates for these additional components of covariation which are measured by the DNAm network correlation measure, beyond gene expression, include other components of biochemical interactive behaviour such as those associated with alternatively spliced forms of the gene product (such alternatively spliced forms are thought to be linked to DNA methylation, [7]). However, it is not possible to test these hypotheses further using the publicly available data analysed here. A further study, involving collection of additional data such as RNA-seq or other transcriptome-level data collected with DNA methylation data, would have the potential for investigating such additional possibilities.

### Association of the DNAm Network Correlation Measure with Patient Survival Outcome

The DNA methylation network correlation measure was tested against patient survival outcome, and further methodology was developed in this context, in order to develop the DNAm network correlation measure as a basis for prognostic biomarkers. For each data set, for each pair of genes, the association of the DNAm network correlation measure with patient overall survival outcome was tested by Cox regression, adjusted for clinical covariates. This adjustment for clinical covariates was carried out in order to develop a novel prognostic measure which is independent of known prognostic clinical features, such as age, disease stage and grade, and to take account of possible confounding (see ‘methods and models’ for further details). Heatmaps showing *p*-values of association of the DNAm network correlation measure with patient survival outcome, for the BRCA data set, for all interactions and for just those which appear in the canonical human interactome map, appear in figure S1, together with equivalent heatmaps produced from randomly generated *p*-values, to show the structure in this network model provided by the canonical human interactome map.

**Figure 2:**
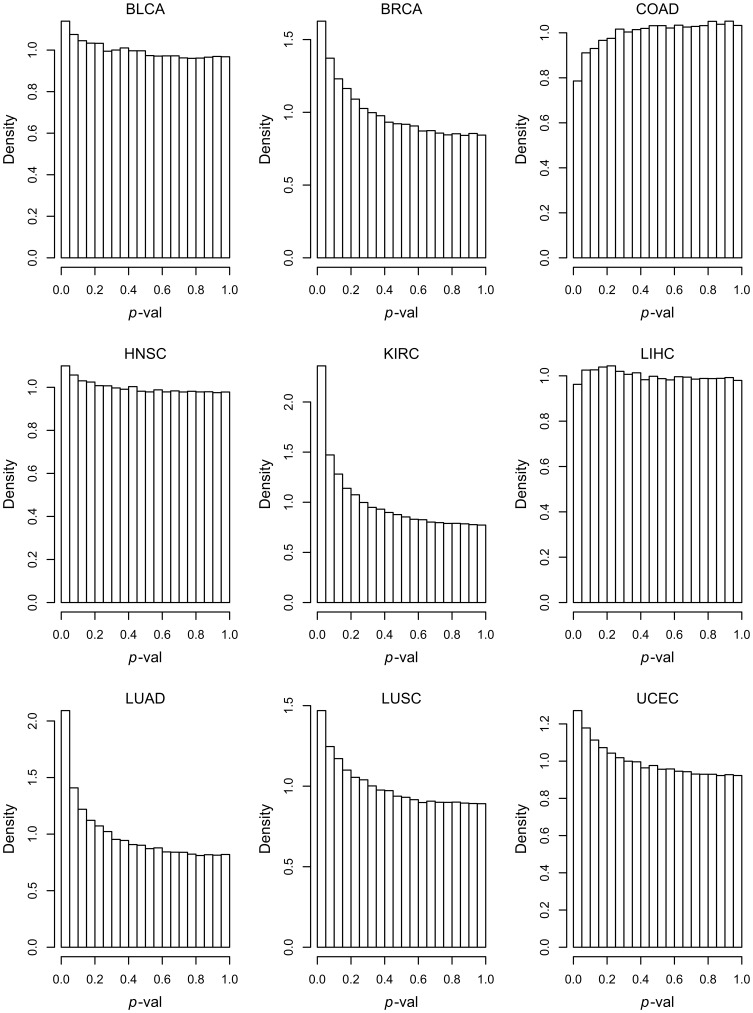
*p*-value histograms, showing the association of the DNA methylation network correlation measure with patient overall survival outcome, for all data sets. For each network edge, which connects a pair of genes (nodes), Cox regression is used to calculate a *p*-value (adjusted for clinical covariates), which represents the association of the DNAm network correlation measure for that edge with patient survival outcome. For COAD and LIHC, there is no association of the DNAm network correlation measure with patient outcome. For the other data sets (cancer types), and in particular BRCA, KIRC, LUAD, LUSC and UCEC, the collection of *p*-values close to 0 shows that, for those data sets, the DNAm network correlation measure for many network edges is associated with patient outcome.

The canonical human interactome map defines 276136 interactions for the 8614 genes which are also present in each of the data sets considered here. For each of the 9 data sets, the 276136 *p*-values resulting from the tests of association with patient overall survival outcome (adjusted for clinical covariates) for each of these interactions are plotted in histograms, which appear in figure 2. For the COAD and LIHC data sets there is no association with survival. However for the other seven data sets (cancer types), and in particular BRCA, KIRC, LUAD, LUSC and UCEC, the concentration of *p*-values close to 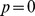 shows that there are many pairs of genes for which the DNAm network correlation measure associates significantly with survival. It is important to note that the poor correlation for certain data sets is strongly linked, as would be expected, to the size of the data sets, and in particular to the number of events in the data sets (table 1). Applying these methods to larger data sets will be necessary for the development and validation of robust biomarkers.

### Identification of Significant and Biologically Relevant Subnetworks

The data sets for which the DNAm network correlation measure did not show a correlation with survival outcome, COAD and LIHC, were excluded from the subsequent analyses, which proceeded with the seven remaining data sets, BLCA, BRCA, HNSC, KIRC, LUAD, LUSC and UCEC. If genes represent network nodes, significant subnetworks, modules, or motifs can be derived from the canonical human interactome map, by retaining the network edges (i.e., pairwise interactions between genes) which correspond to the DNAm network correlation measures which associate significantly with patient overall survival outcome. A significant subnetwork, module, or motif is then made up of genes which are connected by edges which are significantly associated with survival outcome. Some genes are connected to a large number of other genes in the canonical interactome map (i.e., they have high degree). If edges are defined as significant when they correspond to 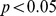 of association with patient survival outcome, then a node (gene) of high degree would be expected to be connected to some other genes by significant edges which are in fact false positives. If several nodes of high degree are connected together by false positives in this way, it would result in a subnetwork being falsely declared significant; this could happen anywhere in the canonical interactome map where several high-degree nodes are connected together. To mitigate this effect, for each node (gene), the *p*-values of association with survival outcome for each of its connected edges (interactions) were converted to FDR (false discovery rate) adjusted *q*-values [13] with respect to that node (gene). Each edge (interaction) was then only marked as significant if it associated significantly with patient survival outcome with 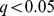 according to both the nodes (genes) connected by that edge.

Significant subnetworks, modules, and motifs found in this way were tested for biological relevance, by checking the genes comprising each subnetwork using gene set enrichment analysis (GSEA) [14]. For each significant subnetwork, module or motif, 6811 gene set definitions, downloaded from the Broad Institute Molecular Signatures Database (http://www.broadinstitute.org), were tested one by one for enrichment by the genes which comprise the respective subnetwork, module, or motif; further details about these gene sets can be found at that website. These enrichment tests were carried out using a one-sided Fisher’s exact test, and if any of the 6811 gene sets showed significant enrichment (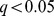) by the genes comprising a subnetwork, module, or motif, it was marked as having biological relevance, according to the findings of previous work. The numbers of significant subnetworks, modules and motifs, arranged according to number of genes/nodes, and the numbers of these found to be biologically relevant, appear in table 3.

**Table 3:** Number of significant subnetworks, modules and motifs.

Nodes	BLCA	BRCA	HNSC	KIRC	LUAD	LUSC	UCEC
3	4 (1)	19 (8)	9 (5)	19 (6)	38 (10)	11 (2)	8 (1)
4	1 (0)	9 (3)	2 (0)	9 (3)	12 (6)	5 (1)	5 (1)
5	2 (2)	1 **(1)**	1 (0)	1 (0)	5 (1)	0 (0)	4 (1)
6	1 (0)	1 (0)	1 (0)	1 (0)	2 (0)	1 (0)	2 (2)
7	0 (0)	1 (0)	1 (0)	1 (1)	2 (0)	0 (0)	0 (0)
8	0 (0)	0 (0)	0 (0)	1 **(1)**	2 (0)	0 (0)	1 (1)
9	1 **(1)**	1 (0)	0 (0)	2 (0)	1 (1)	1 (0)	0 (0)
10	0 (0)	0 (0)	0 (0)	0 (0)	0 (0)	1 (0)	0 (0)
11	0 (0)	0 (0)	1 (0)	0 (0)	1 (1)	0 (0)	2 **(1)**
13	0 (0)	0 (0)	0 (0)	0 (0)	1 **(1)**	0 (0)	0 (0)
14	0 (0)	0 (0)	0 (0)	0 (0)	0 (0)	1 (0)	0 (0)
16	0 (0)	0 (0)	0 (0)	0 (0)	0 (0)	1 (0)	0 (0)
17	1 (0)	0 (0)	0 (0)	0 (0)	0 (0)	0 (0)	0 (0)
18	0 (0)	0 (0)	0 (0)	0 (0)	0 (0)	1 **(1)**	0 (0)
24	1 (0)	0 (0)	0 (0)	0 (0)	0 (0)	0 (0)	0 (0)
27	0 (0)	0 (0)	1 (0)	0 (0)	0 (0)	0 (0)	0 (0)
373	0 (0)	0 (0)	0 (0)	0 (0)	1 **(1)**	0 (0)	0 (0)
770	0 (0)	0 (0)	0 (0)	1 **(1)**	0 (0)	0 (0)	0 (0)

**Figure 3:**
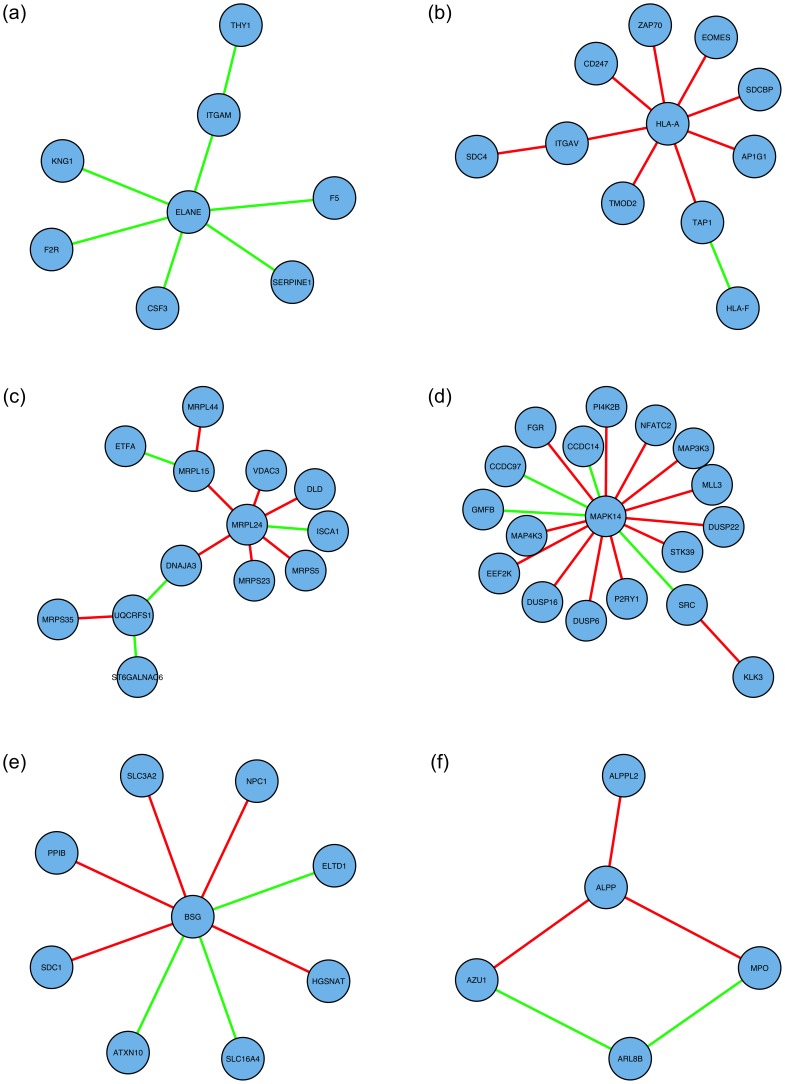
Smaller significant network modules: network diagrams. Network edges displayed in green and red indicate positive and negative hazard ratios, respectively, for the DNAm network correlation measure corresponding to that interaction; these correspond, respectively, to an increase and decrease in ‘network interaction co-ordinatedness’ for worse disease prognosis. (a) Wound healing module (KIRC). (b) Immune module (UCEC). (c) Mitochondial module (LUAD). (d) MAP-kinase module (LUSC). (e) Largest biologically significant subnetwork in the BLCA data set. (f) Largest biologically significant subnetwork in the BRCA data set. Further details about the corresponding network nodes (genes) and significantly enriched gene sets appear in tables 6–9 and S1–4.

**Figure 4:**
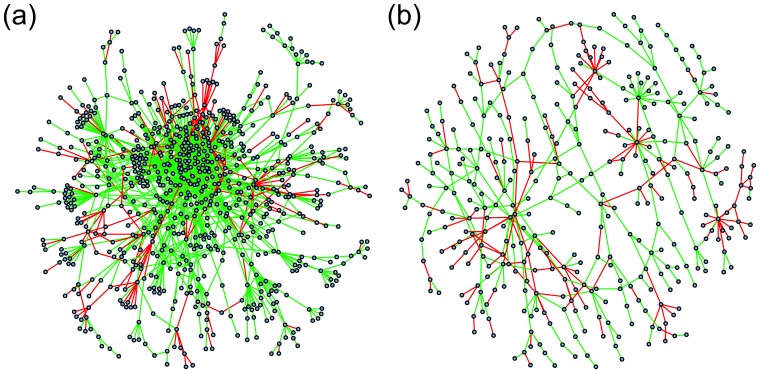
Larger significant subnetworks: network diagrams. Network edges displayed in green and red indicate positive and negative hazard ratios, respectively, for the DNAm network correlation measure corresponding to that interaction; these correspond, respectively, to an increase and decrease in ‘network interaction co-ordinatedness’ for worse disease prognosis. (a) the KIRC large subnetwork. (b) the LUAD large subnetwork. Further details about the corresponding network nodes (genes) for the top 5% of the degree distribution and top 25 significantly enriched gene sets appear in tables S5–6.

The number of significant subnetworks, modules and motifs found with each number of nodes (genes) is shown for each data set. Of these, the numbers found to have biological relevance (as determined by gene set enrichment analysis) are shown in brackets. Modules and larger subnetworks subsequently plotted in figures 3 and 4 are shown in bold.

To check how well this methodology guards against falsely declaring subnetworks as significant, exactly the same process of significant subnetwork identification was applied to *p*-values randomly generated by sampling from a uniform(0,1) distribution and assigned to the 276136 interactions, and this process was repeated 10^4^ times. Table 4 shows the number of null subnetworks produced, of each size, in total over the 10^4^ iterations. If there were no association of the DNAm network correlation measure with survival outcome (which would correspond to uniformly distributed *p*-values across these features), the expected number of 3-node subnetworks falsely declared as significant in a particular data set would be 4.0, the expected number of falsely identified 4-node subnetworks would be 0.34, and for 5 nodes the expected number would be 0.037. Comparison of these numbers with table 3 and the definition of the false discovery rate implies (taking into account these seven data sets) 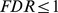 for 3 node subnetworks, 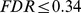 for 4 node subnetworks, and 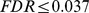 for 5 node subnetworks. Further, no individual gene appeared in these null subnetworks with at least 5 nodes more than twice out of the 10^4^ iterations. As these small motifs of 3 and 4 nodes are of less interest in general, and because by excluding them the subnetwork false discovery rate is well-controlled, in the subsequent analysis, only subnetworks, modules and motifs with at least 5 nodes were considered as significant.

**Table 4:** Null subnetwork analysis.

No. nodes	No. null subnetworks	Expected false positivesubnets per dataset	Min no. subnetsper dataset	Max *FDR*
3	40124	4.0	4	1
4	3392	0.34	1	0.34
5	365	0.037	1	0.037
6	48	0.0048	1	0.0048
7	4	4×10^−4^	1	4×10^−4^
8	1	1×10^−4^	1	1×10^−4^

10^4^ iterations of null subnetworks were generated, by the same subnetwork identification method as used for the real data sets, but based on *p*-values randomly sampled from a uniform distribution. The table shows the number of subnetworks of each size which were declared as significant by the subnetwork identification method based on these null *p*-values, out of the 10^4^ iterations. The table also shows the minimum number of subnetworks of each size detected in any of the real data sets (see table 3), and corresponding conservative estimates of the *FDR*, defined as the number of false positives divided by the number of discoveries, for each size of subnetwork.

A network edge which is significant due to a correlation between a higher value of the DNAm network correlation measure and worse patient survival time (i.e., hazard ratio, 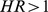), corresponds to an increasing tendency of the genes (nodes) at either end of this network edge to explain each other’s regulatory behaviour, the worse the prognosis of the cancer. This can be thought of as an increase in network interaction ‘co-ordinatedness’ between these genes corresponding to worse disease prognosis, or a ‘positive network re-wiring’ that is adaptively advantageous for the cancer. The opposite effect, where a network edge is significant due to a correlation between a lower value of the DNAm network correlation measure and worse patient survival time (i.e., hazard ratio, 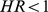), is equivalent to a decrease in network interaction co-ordinatedness for worse disease prognosis, or ‘negative network re-wiring’. The proportion of significant network edges with 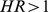 (increase in network interaction co-ordinatedness for worse disease prognosis) is shown for each data set in table 5.

**Table 5:** Directionality of significant network edges.

Data set	Prop. *HR*>1
BLCA	0.32
BRCA	0.46
HNSC	0.47
KIRC	0.82
LUAD	0.66
LUSC	0.66
UCEC	0.37

The numbers in the second column indicate the proportion of network edges (i.e., pairs of genes) which increase (rather than decrease) their ‘network interaction co-ordinatedness’ for worse disease prognosis (i.e., the DNAm network correlation measure increases for shorter typical patient survival time, 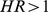).

### Smaller Significant and Biologically Relevant Network Modules of Interest

Detailed consideration of the smaller network modules identified as significant, and biologically relevant, confirms their relevance to cancer biology. Amongst the gene sets significantly enriched by the significant network modules, as well as numerous specific cancer gene sets, gene sets appear multiple times relating to JAK-STAT signalling, WNT signalling, GPCR signalling, EGFR signalling, VEGF signalling, interleukin activity, neutrophil activity/response to wounding, immune activity, metabolic activity/TCA cycle/mitochondria, chromosome maintenance, developmental processes/stem cells, programmed cell death, response to UV/DNA damage repair/Fanconi anemia, cell cycle, transcriptional regulation, transcriptional activity, and transport/trafficking.

Six examples of smaller network modules identified as significant and biologically relevant are shown in figure 3; these include modules related to wound healing (associated with cancer invasion and progression to new sites), immune function (associated with stifling the body’s ability to fight the cancer, mitochondrial function (associated with increased energy production required for cancer proliferation) and MAP-kinase signalling (associated with regulation of cell proliferation). Summaries of the genes/nodes which appear in these network modules, and of the respective significantly enriched gene sets, appear in tables 6 and 7 (wound healing), tables 8 and 9 (immune), table S1 (mitochondrial), table S2 (MAP-kinase), and tables S3 and S4 (largest biologically significant subnetworks found in the BLCA and BRCA datasets, respectively).

**Table 6:** Wound-healing module: gene/node details.

Gene/node	Degree	Chr	Gene info
ELANE	6	19	elastase, neutrophil expressed
ITGAM	2	16	integrin, alpha M (complement component 3 receptor 3 subunit)
CSF3	1	17	colony stimulating factor 3 (granulocyte)
F2R	1	5	coagulation factor II (thrombin) receptor
F5	1	1	coagulation factor V (proaccelerin, labile factor)
KNG1	1	3	kininogen 1
SERPINE1	1	7	serpin peptidase inhibitor, clade E (nexin, plasminogen activator inhibitor type 1), member 1
THY1	1	11	Thy-1 cell surface antigen

Gene/node details for the wound healing module found as significant in the KIRC data set.

**Table 7:** Wound-healing module: significantly enriched gene sets.

Gene set	OR (95% C.I.)	*q*-val
BLOOD_COAGULATION	290 (52–1600)	5.3e-05
COAGULATION	290 (52–1600)	5.3e-05
HEMOSTASIS	250 (46–1500)	5.7e-05
WOUND_HEALING	230 (42–1300)	5.9e-05
REGULATION_OF_BODY_FLUID_LEVELS	210 (37–1100)	7e-05
KEGG_COMPLEMENT_AND_COAGULATION_CASCADES	200 (36–1100)	7e-05
BIOCARTA_INTRINSIC_PATHWAY	450 (63–2600)	0.00023
NEGATIVE_REGULATION_OF_MULTICELLULAR_ORGANISMAL_PROCESS	320 (45–1800)	0.00054
WANG_ESOPHAGUS_CANCER_VS_NORMAL_UP	100 (19–580)	0.00057
PID_INTEGRIN2_PATHWAY	230 (34–1300)	0.001
REGULATION_OF_BIOLOGICAL_QUALITY	50 (9.7–320)	0.0014
RESPONSE_TO_WOUNDING	69 (13–380)	0.0019
PID_UPA_UPAR_PATHWAY	170 (25–940)	0.0019
REACTOME_HEMOSTASIS	41 (7.9–260)	0.003
REACTOME_PLATELET_ACTIVATION_SIGNALING_AND_AGGREGATION	54 (9.9–290)	0.0044
BIOCARTA_FIBRINOLYSIS_PATHWAY	680 (53–8200)	0.006
RESPONSE_TO_EXTERNAL_STIMULUS	42 (7.8–230)	0.0092
REACTOME_RESPONSE_TO_ELEVATED_PLATELET_CYTOSOLIC_CA2_	86 (13–450)	0.0092
BIOCARTA_GRANULOCYTES_PATHWAY	460 (38–4100)	0.0094
RECEPTOR_BINDING	35 (6.6–190)	0.016
BIOCARTA_EXTRINSIC_PATHWAY	310 (27–2200)	0.017
REGULATION_OF_MULTICELLULAR_ORGANISMAL_PROCESS	52 (7.9–270)	0.032
RPS14_DN.V1_UP	47 (7.2–250)	0.041

Significantly enriched gene sets, for the wound healing module found as significant in the KIRC data set. *Q*-values indicate significance of enrichment in the corresponding gene set by the genes in this module, calculated according to a one-sided Fisher’s exact test. Further details about these gene sets can be found from the website of the Broad Institute Molecular Signatures Database (http://www.broadinstitute.org).

**Table 8:** Immune module: gene/node details.

Gene/node	Degree	Chr	Gene info
HLA-A	8	6	major histocompatibility complex, class I, A
ITGAV	2	2	integrin, alpha V
TAP1	2	6	transporter 1, ATP-binding cassette, sub-family B (MDR/TAP)
AP1G1	1	16	adaptor-related protein complex 1, gamma 1 subunit
CD247	1	1	CD247 molecule
EOMES	1	3	eomesodermin
SDCBP	1	8	syndecan binding protein (syntenin)
TMOD2	1	15	tropomodulin 2 (neuronal)
ZAP70	1	2	zeta-chain (TCR) associated protein kinase 70 kDa
HLA-F	1	6	major histocompatibility complex, class I, F
SDC4	1	20	syndecan 4

Gene/node details for the immune module found as significant in the UCEC data set.

**Table 9:** Immune module: significantly enriched gene sets.

Gene set	OR (95% C.I.)	*q*-val
REACTOME_ADAPTIVE_IMMUNE_SYSTEM	37 (9.3–170)	0.001
REACTOME_ANTIGEN_PROCESSING_CROSS_PRESENTATION	97 (20–390)	0.0019
REACTOME_ANTIGEN_PRESENTATION_FOLDING_ASSEMBLY_AND_PEPTIDE_LOADING_OF_CLASS_I_MHC	210 (33–980)	0.0036
REACTOME_THE_ROLE_OF_NEF_IN_HIV1_REPLICATION_AND_DISEASE_PATHOGENESIS	140 (23–670)	0.006
REACTOME_IMMUNE_SYSTEM	22 (5.5–100)	0.006
KEGG_CELL_ADHESION_MOLECULES_CAMS	52 (11–210)	0.0069
GNF2_HLA-C	110 (17–480)	0.0094
GNF2_INPP5D	100 (17–460)	0.0094
GNF2_ITGAL	100 (16–440)	0.0094
REACTOME_IMMUNOREGULATORY_INTERACTIONS_BETWEEN_A_LYMPHOID_AND_A_NON_LYMPHOID_CELL	89 (15–390)	0.012
RECEPTOR_COMPLEX	76 (13–330)	0.013
GNF2_CD53	80 (13–350)	0.013
KEGG_ANTIGEN_PROCESSING_AND_PRESENTATION	76 (13–330)	0.013
PID_CD8TCRDOWNSTREAMPATHWAY	80 (13–350)	0.013
PID_CD8TCRPATHWAY	71 (12–310)	0.014
REACTOME_ER_PHAGOSOME_PATHWAY	71 (12–310)	0.014
REACTOME_ENDOSOMAL_VACUOLAR_PATHWAY	370 (32–2500)	0.015
DER_IFN_ALPHA_RESPONSE_UP	66 (11–290)	0.015
PID_IL12_2PATHWAY	65 (11–280)	0.015
BIOCARTA_TCRA_PATHWAY	310 (27–2200)	0.015
REACTOME_NEF_MEDIATED_DOWNREGULATION_OF_MHC_CLASS_I_COMPLEX_CELL_SURFACE_EXPRESSION	310 (27–2200)	0.015
REACTOME_TRANSLOCATION_OF_ZAP_70_TO_IMMUNOLOGICAL_SYNAPSE	310 (27–2200)	0.015
DER_IFN_GAMMA_RESPONSE_UP	61 (10–270)	0.015
BIOCARTA_CTL_PATHWAY	270 (24–1600)	0.018
MODULE_293	270 (24–1600)	0.018
REACTOME_CLASS_I_MHC_MEDIATED_ANTIGEN_PROCESSING_PRESENTATION	27 (5.8–110)	0.018
IMMUNOLOGICAL_SYNAPSE	230 (21–1400)	0.021
MODULE_143	210 (19–1200)	0.025
GNF2_MATK	140 (14–820)	0.043
KEGG_NATURAL_KILLER_CELL_MEDIATED_CYTOTOXICITY	38 (6.4–160)	0.043
SENGUPTA_EBNA1_ANTICORRELATED	38 (6.4–160)	0.043
GNF2_ZAP70	130 (13–740)	0.045
BIOCARTA_CSK_PATHWAY	130 (13–740)	0.045

Significantly enriched gene sets, for the immune module found as significant in the UCEC data set. *Q*-values indicate significance of enrichment in the corresponding gene set by the genes in this module, calculated according to a one-sided Fisher’s exact test. Further details about these gene sets can be found from the website of the Broad Institute Molecular Signatures Database (http://www.broadinstitute.org).

Importantly, the network interactions in the wound healing module predominantly show an increase in network interaction co-ordinatedness with worse disease prognosis, indicating a tendency towards co-ordinated behaviour of these genes in support of metastatic processes. Conversely, the network interactions in the immune subnetwork module predominantly show a decrease in network interaction co-ordinatedness with worsening disease prognosis, suggesting a degradation of the body’s own defences against the tumour. Hence, the DNA methylation network correlation measure, as a surrogate measure of more general interactive behaviour of genes, reflects increases and decreases in interactive behaviour in subnetwork modules which would be expected according to their biological function and role in disease.

The genes which comprise the wound healing module, and an outline of their biological roles, are as follows. ELANE is neutrophil elastase; it is secreted by neutrophils and macrophages during inflammation, and destroys bacteria and host tissue [15]. KNG1 is kininogen 1, which uses alternative splicing to generate two different proteins, including HMWK which is important for coagulation of blood [16]. SERPINE1 encodes the serpin peptidase inhibitor, which plays a key role in the inhibition of fibrinolysis (the physiological breakdown of blood clots) [17]. F2R is proteinase-activated receptor 1, which is involved in the regulation of thrombotic response (clotting in blood vessels) [18]. F5 is factor five, a protein of the coagulation system; its deficiency leads to predisposition for haemorrhage [19]. CSF3 is granulocyte colony-stimulating factor, which promotes the differentiation and proliferation of blood cells [20]. ITGAM is integrin alpha M, which mediates inflammation by regulating leukocyte adhesion and migration [21]. THY1 is cluster of differentiation 90, which amongst other functions has a role in cell adhesion and migration [22].

The genes which comprise the immune module, and an outline of their biological roles, are as follows. HLA-A is a human leukocyte antigen (HLA); HLA constitute a large subset of the major histocompatibility complex (MHC, which mediates the interaction of immune system white blood cells) [23]. ITGAV is the vitronectin receptor integrin (integrins mediate attachment between a cell and its surroundings); vitronectin is found in serum and the extracellular matrix (ECM) and has been implicated in tumour malignancy [24]. SDC4 is syndecan 4, which interacts with ECM, anticoagulants, and growth-factors, and regulates the actin cytoskeleton, cell adhesion, and cell migration [25]. SDCBP is syndecan binding protein, which is thought to function as an adaptor protein, coupling the important developmental/pluripotency transcription factor SOX4 to the interleukin-5 receptor (which stimulates immune B-cell growth) [26]. CD247 encodes a component of the zeta-chain, which is part of the immune T-cell surface antigen receptor (TCR), which serves antigen recognition and signalling functions [27]. TMOD2 is a tropomodulin specific to neurons; tropomodulins cap the ends of actin filaments [28]. TAP1 is ‘transporter associated with antigen processing, involved with transporting molecules across extra and intra cellular membranes, associated with the MHC [29]. HLA-F is another human leukocyte antigen (component of the MHC). ZAP-70 is zeta-chain-associated protein kinase 70, another component of the TCR [30]. EOMES is eomesodermin, which encodes a transcription factor which is thought to be necessary for the differentiation of effector CD8+ T cells [31]. AP1G1 is AP-1 complex subunit gamma-1, which has a role in promoting the formation of clathrin-coated pits and vesicles, which are used to transport molecules within and between cells [32].

### Larger Significant Subnetworks

**Figure 5:**
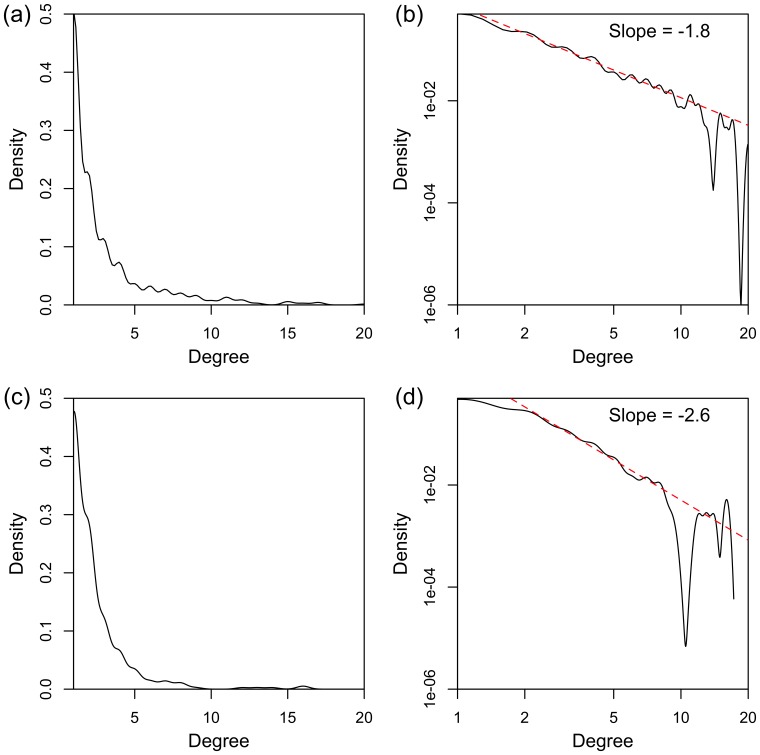
Larger significant subnetworks: degree distributions. Degree distributions (kernel-smoothed) on linear and log scales, for (a), (b) KIRC large subnetwork, and (c), (d) LUAD large subnetwork. Dashed red lines on the log-log plots display the power-law linear model line of best fit, estimated from the nodes with degree in the range 2 to 8, with the corresponding slope value (i.e., power law exponential coefficient) displayed above.

In each of two data sets, KIRC and LUAD, a larger significant subnetwork was also found (with 770 and 373 nodes respectively), and these are shown in figure 4; the higher density of nodes in the case of the KIRC large subnetwork is clearly seen in this figure. Both these large subnetworks were found to be biologically relevant, with 474 and 156 gene sets respectively found as significant by GSEA (). Degree distributions on a linear, and on a log-log scale, are shown in figure 5; there is power-law type behaviour in both cases for degree in the range 2 to 8 (linear model lines estimating the logarithm of the degree distribution, with corresponding slope estimates, are shown in the figure).

Considering the *p*-value histograms in figure 2, there are many more significant *p*-values for KIRC and LUAD than the other data sets, as can be seen by the height of the histogram bars closest to 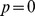, and correspondingly more significant network edges for these data sets. This may be why these data sets are the only ones for which such large subnetworks are found. Most of the data sets considered in this study have small sample sizes, particularly with respect to the number of events, and hence relatively low statistical power. If these methods were applied to larger data sets, larger subnetworks such as these might be able to be found for other cancer types. The degree density also decreases less as degree increases in the case of KIRC (figure 5 (b), slope = −1.8) than in the case of LUAD (figure 5 (b), slope = −2.6). This is as might be expected given that there are more significant network edges in the case of KIRC than LUAD (figure 2). This has the implication that LUAD shows fewer nodes of very high numbers of connections, so called “hubs” (see also tables S5–6). The presence of “hubs” is related to the probability of network failure. If a few nodes control the connectivity of the whole network, then their elimination will make the network fail. If the degree distribution is more even, and thus the slope less steep, then the network may be more resilient to failure. The slope is therefore an important characteristic of the network.

One feature of interest in relation to these two larger significant subnetworks is that they contain a high proportion of pairs of genes (i.e., network edges) which increase their ‘network interaction co-ordinatedness’, the worse the prognosis of the cancer. In fact, this is a characteristic of the KIRC and LUAD data sets particularly, although not of all the data sets (table 5). This could be another reason why such large subnetworks are found for these data sets. Whereas interactions which correspond to a decrease in interactive behaviour with disease progression might logically correspond to fragmented, smaller motifs and modules, interactions which correspond to an increase in interactive behaviour with disease progression might be expected to coalesce to form larger subnetworks, with a tendency to act more autonomously.

Details about the genes/nodes in the top 5% of the degree distribution (as a summary of the most significant nodes) and the 25 most significantly enriched gene sets are shown in table S5 (KIRC) and table S6 (LUAD). It is particularly noticeable that there is enrichment by the genes in the significant large subnetworks of many genes sets associated with transcriptional and translational processes, and perhaps the most interesting of these in the context of DNAm relate to splicing. For example, the splicing factor gene SF3B4 is the node with the second highest degree in the KIRC large subnetwork (table S5 (a)), and the KEGG spliceosome gene set is very significantly enriched by the nodes of the KIRC large subnetwork, 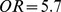 (95% C.I. 3.5–9.2), FDR-adjusted 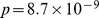 (Fishers exact test), table S5 (b). DNA methylation has been suggested to have an important association with alternative splicing [33], although how this might work is poorly understood. It is very interesting that the largest significant subnetworks identified by this method appear to be involved in these processes, amongst other forms of gene regulatory behaviour, and more generally cancer-related processes.

## Discussion

A DNA methylation (DNAm) measure of network correlation has been developed, as a measure of ‘network interaction co-ordinatedness’, between pairs of genes, in terms of their DNAm profiles. This measure has been shown to be highly associated with the correlation of gene expression measurements (at the mRNA level) from the same pairs of genes in five independent data sets from different cancer types; i.e., the DNAm network correlation measure reflects well co-regulatory behaviour relating to gene expression. This measure has been tested for association with patient overall survival outcome independent of known clinical prognostic features in nine independent data sets corresponding to different cancer types; independent association with survival outcome was found for this measure in seven out of nine of these data sets, with strong association in five of these, which are Breast Invasive Carcinoma (BRCA), Kidney Renal Clear Cell Carcinoma (KIRC), Lung Adenocarcinoma (LUAD), Lung Squamous Cell Carcinoma (LUSC), and Uterine Corpus Endometrioid Carcinoma (UCEC). For each of these data sets (cancer types), significant subnetworks, modules, and motifs have been found, with the associated false discovery rate shown to be well-controlled for those with at least 5 nodes (genes). In many cases, these significant subnetwork modules are shown to have strong correlation with groups of genes previously found to be biologically relevant, in the context of cancer biology.

The smaller significant subnetwork modules identified in relation to previous knowledge of cancer biology include ones identified with wound healing (associated with cancer invasion and progression to new sites), mitochondrial function (associated with increased energy production required for cancer proliferation), immune function (associated with stifling the body’s ability to fight the cancer), and MAP-kinase signalling (associated with regulation of cell proliferation). The wound healing module interactions show an increase in network co-ordinatedness with worsening disease prognosis, indicating a tendency towards a re-wiring of this module in support of metastatic processes. The immune module interactions show a decrease in network co-ordinatedness with worsening disease prognosis, suggesting a degradation of the body’s own defences against the tumour. Larger significant subnetworks found are associated, amongst other things, with functions related to DNA transcription, translation and regulation. These functions notably include those related to splicing, which is of particular interest in relation to current DNAm research.

Wound healing is an example of a biological function which behaves aberrantly in the context of cancer biology, highlighted in a recent paper [34] which fundamentally shifts the paradigm of oncogenesis. Those authors suggest that tumourigenic processes are actually a regression to archaic metazoan phenotypic characteristics normally suppressed in healthy tissue, which correspond to groups of cells behaving more autonomously. The key point in this metazoan model is that, whereas the conventional model of tumourigenesis holds that proliferative characteristics acquired by cancers occur as a result of random genetic and epigenetic mutation, these archaic metazoan characteristics are present in humans all along, but lie dormant until they are released in cancer. The data considered in this study does not allow this point to be addressed directly, but intuitively it seems that our findings fit well into this new metazoan oncogenic paradigm.

The quality of the presented study depends on the availability of clinical data. However, information is missing for many samples for several clinical covariates. Also, for a number of clinical covariates, the large majority of the samples are in the same category for that covariate. It should therefore be expected that this analysis will not have fully taken account of some clinical covariates. Further, the clinical information considered does not include descriptions of applied therapies. For these reasons, more detailed analyses relating to specific cancers should be expected to be necessary for the further development of clinical biomarkers based on the methods developed here.

While the data sets considered in this study do not contain enough events to train a predictive model, bigger data sets would allow such predictive prognostic models to be defined using these methods, leading to novel independent DNAm-based disease prognostic biomarkers. Such biomarkers would be based on DNA rather than RNA, and would be ideal for further investigation and development using current state-the-art and future research findings from the rapidly advancing field of network science.

## Methods and Models

### Data Download and Preprocessing

DNA methylation (DNAm) data, collected via the Illumina Infinium HumanMethylation450 platform, were downloaded from The Cancer Genome Atlas (TCGA) project [9]–[11] at level 3. These data were obtained for nine different tumour types, as follows: Bladder Urothelial Carcinoma (BLCA), Breast Invasive Carcinoma (BRCA), Colon Adenocarcinoma (COAD), Head and Neck Squamous Cell Carcinoma (HNSC), Kidney Renal Clear Cell Carcinoma (KIRC), Liver (LIHC), Lung Adenocarcinoma (LUAD), Lung Squamous Cell Carcinoma (LUSC), and Uterine Corpus Endometrioid Carcinoma (UCEC).

These data were pre-processed by first removing probes with non-unique mappings and which map to SNPs (as identified in the TCGA level 3 data); probes mapping to sex chromosomes were also removed; in total 98384 probes were removed in this way from all data sets. After removal of these probes, 270985 probes with known gene annotations remained. Individually for each data set, probes were then removed if they had less than 95% coverage across samples; probe values were also replaced if they had corresponding detection *p*-value greater than 5%, by KNN (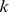 nearest neighbour) imputation (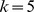). The loci of analysed CpGs were mapped to genes based on annotation information for the Illumina Infinium platform obtained from the R [35]/Bioconductor [36] package ‘IlluminaHumanMethylation450 k’. The data were also checked for batch effects by hierarchical clustering and correlation of the significant principle components with phenotype and batch: no significant batch effects (which would warrant further correction) were found.

A list of pairs of genes, each pair corresponding to a known biochemical interaction in humans, was downloaded from http://www.pathwaycommons.org, and was used as a canonical human interactome map in the subsequent analysis. In this network model, each pair of genes defines a network edge (422481 edges in total), with individual genes represented by network nodes (12726 nodes in total).

For each data set/cancer type, clinical information was also downloaded, for the variables overall survival status (alive or not), overall survival time (i.e., time to last follow up or time to death), as well as several clinical covariates for each data set, such as age, disease stage (I–IV), disease grade (1–3), and residual disease status (present or not). All survival analyses were carried out using the R [35] package ‘survival’.

For five of the cancer types (BRCA, COAD, KIRK, LUSC and UCEC), gene expression data were also downloaded from TCGA at level 3. These data were quantile-normalised.

### DNA Methylation Network Correlation Measure Definitions

Canonical correlation analysis (CCA) [8] seeks to find the vectors 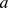 and 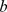, in the 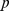 and 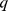 dimensional spaces of variables 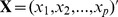 and 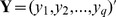 respectively, which maximise the correlation 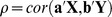, defined according to equation 1,
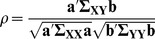
(1)where 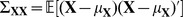 and 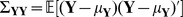 are the covariance matrices of 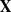 and 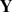 respectively, and 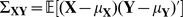 is the cross-covariance matrix of 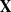 and 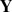.

Two genes 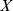 and 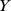, which are joined by a network edge in the interactome map (i.e., these genes or their products participate in some canonical biochemical interaction), have corresponding methylation profiles which are measured for sample/patient 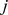 at 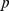 and 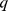 CpGs (loci) respectively along these genes. Denoting these measurements by the variables 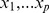 and 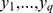 for genes 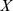 and 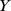 respectively, the DNA methylation profiles for these genes, for patient 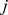, can be represented by the vectors 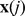 and 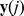, which have 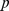 and 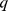 entries respectively. A measure of DNAm network correlation 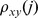, of the methylation profiles of genes 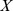 and 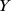 for sample 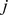, can then be defined by analogy with equation 1, according to equation 2,
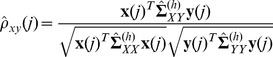
(2)where 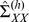, 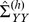 and 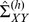 are estimated from healthy rather than cancer samples in the methylation data set, according to equations 3–5,



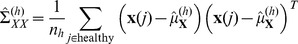
(3)

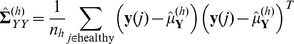
 (4)
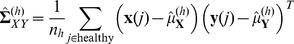
(5)where



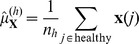
and



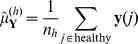
and 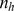 is the number of healthy samples in the data set.

### Selection of Significant Clinical Covariates

If a clinical covariate (e.g., age, disease stage and grade, or presence of residual disease) associates significantly with the patient outcome of interest (here, overall survival time) and also with the predictors of interest (here, the DNAm network correlation measure), then any association between the predictors and outcome of interest might be due only to the variation of the clinical covariate(s). In order to develop novel DNA methylation biomarkers which are independent of known prognostic clinical features, the statistical tests of association with patient survival outcome are adjusted here for the clinical covariates which also associate significantly with patient survival outcome.

For each data set (corresponding to a particular cancer type), the available clinical covariates were tested one by one for association with overall survival outcome, by fitting a Cox model. For each data set, the covariates for which this association corresponded to 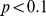 were then considered further, in a multivariate Cox model. Because clinical covariates may be measuring correlated physiological quantities (e.g., height and weight, or in some cases disease stage and grade), it is only necessary to adjust for a clinical covariate in the subsequent DNAm survival analysis if that clinical covariate associates significantly with survival after adjustment for the other clinical covariates. Therefore, the clinical covariates found to be significant in the first stage (by testing their association one by one with with overall survival outcome) were tested against each other in a multivariate Cox regression, and those which remained significant (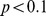) after adjustment for the other clinical covariates were then adjusted for in the subsequent DNAm survival analysis.

For each data set, the clinical covariates which were considered are as follows, with those ultimately found to associate with overall survival (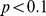), and thus adjusted for in the DNAm survival analysis, shown in bold.

- **BLCA:** **stage**, age, grade, diagnosis subtype, anatomic organ subdivision, height, prior diagnosis, gender, tobacco history, weight.
- **BRCA:** **age**, **residual disease**, stage, gender, ER status, PR status.
- **COAD:** age, stage, prior diagnosis, residual disease, anatomic site, histology, polyps, lymphatic invasion, perineural invasion, gender, height, weight.
- **HNSC:** **residual disease**, **tobacco history**, age, stage, grade, prior diagnosis, pack years, lymph node presentation, gender.
- **KIRC:** **age**, **stage**, **prior diagnosis**, **residual disease**, grade, haemoglobin, platelet, lymph node, calcium, gender.
- **LIHC:** **creatinine**, **prothrombin**, age, stage, grade, prior diagnosis, residual disease, prospective tissue collection, platelet, albumin, alpha fetoprotein, fibrosis, gender, height, weight.
- **LUAD:** **residual disease**, age, stage, tobacco history, prior diagnosis, gender.
- **LUSC:** **residual disease**, age, stage, tobacco history, gender.
- **UCEC:** **stage**, **residual disease**, age, grade, prior diagnosis, height, weight.

## Supporting Information

Figure S1 ***p*** **-value heatmaps, showing the association of the DNAm network correlation measure with patient overall survival outcome, for the BRCA data set.** For the BRCA data set, for each network edge (DNAm network interaction correlation measure), the Cox regression *p*-value (adjusted for clinical covariates) of association with patient survival outcome is displayed according to the colour scale shown on the right. (a) *p*-values are calculated for every possible pair of genes of the 14800 available in this data set, with genes clustered along the margins of the plot using these *p*-values as a distance measure. (b) Null *p*-values are generated by sampling from a uniform distribution bounded on [0,1] for every possible pair of genes, with genes similarly clustered along the margins. (c) and (e) *p*-values are calculated for the 8614 genes which appear in this data set and also in the pathway commons interactome map, for the 276136 interactions between pairs of these genes defined by this interactome map. Genes are similarly clustered along the margins of the plot according to *p*-value. (e) Shows a zoomed-in view of the top-left of (c). (d) and (f) are as (c) and (e), but based on null *p*-values randomly sampled from a uniform (0,1) distribution, to demonstrate the structure present from the pathway commons interactome map, without the influence of the DNA methylation network interaction measure. Pearson correlation coefficients comparing values in these adjacency matrices as plotted, are as follows: (a) vs. (b), 0.0011; (c) vs. (d), 0.26; (e) vs. (f), 0.38.
(JPG)

Table S1 **Mitochondrial module.**
(PDF)

Table S2 **MAP-kinase module.**
(PDF)

Table S3 **Largest biologically significant module, BLCA.**
(PDF)

Table S4 **Largest biologically significant module, BRCA.**
(PDF)

Table S5 **KIRC large subnetwork.**
(PDF)

Table S6 **LUAD large subnetwork.**
(PDF)
