# Supplementary figures and images for: A DNA Methylation Network Interaction Measure, and Detection of Network Oncomarkers
Source: PLoS One. 2014 Jan 6;9(1):e84573. doi: 10.1371/journal.pone.0084573 (PMC3882261; doi:10.1371/journal.pone.0084573)

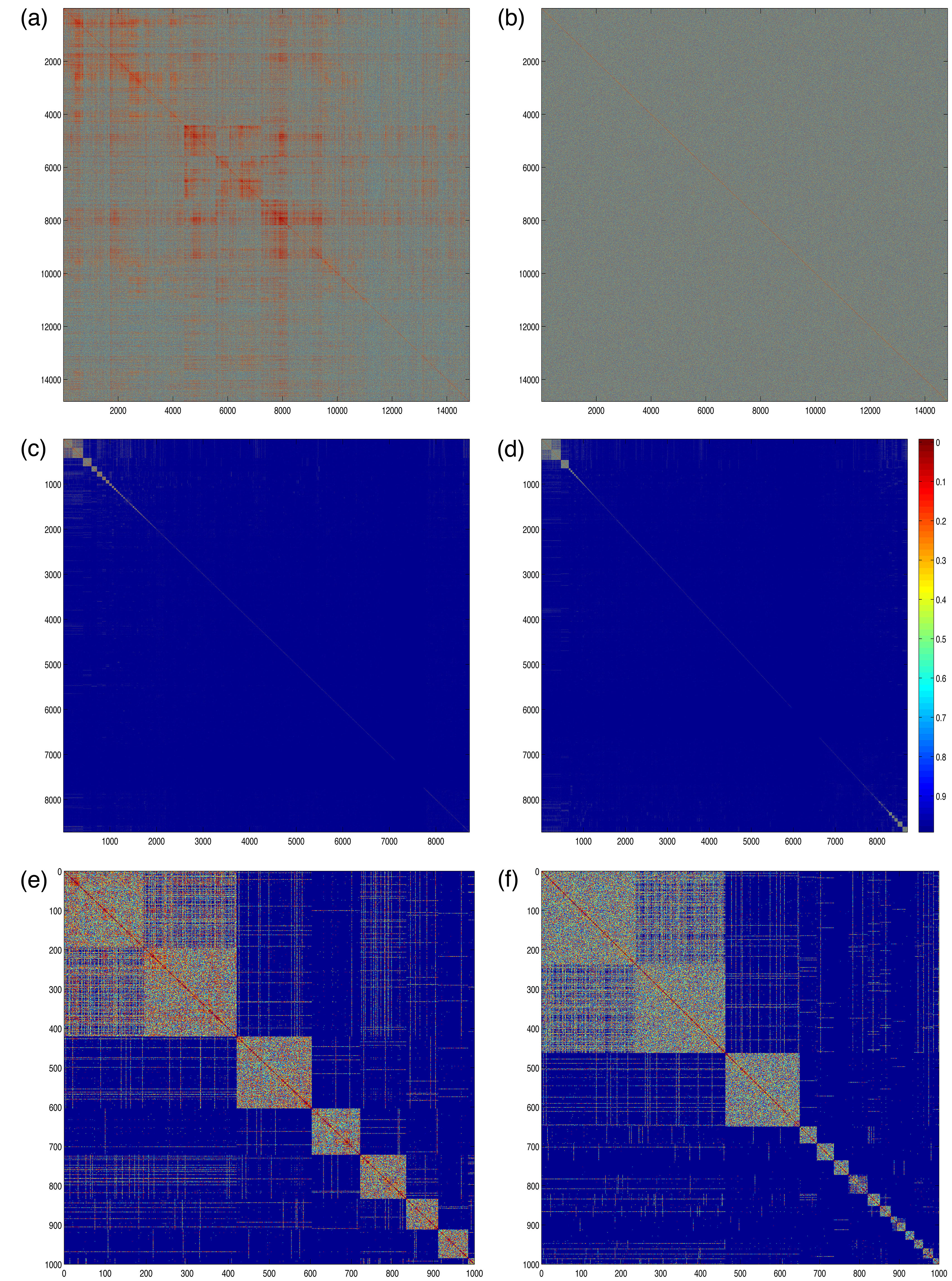

Supplement: Figure S1 — p -value heatmaps, showing the association of the DNAm network correlation measure with patient overall survival outcome, for the BRCA data set. For the BRCA data set, for each network edge (DNAm network interaction correlation measure), the Cox regression p-value (adjusted for clinical covariates) of association with patient survival outcome is displayed according to the colour scale shown on the right. (a) p-values are calculated for every possible pair of genes of the 14800 available in this data set, with genes clustered along the margins of the plot using these p-values as a distance measure. (b) Null p-values are generated by sampling from a uniform distribution bounded on [0,1] for every possible pair of genes, with genes similarly clustered along the margins. (c) and (e) p-values are calculated for the 8614 genes which appear in this data set and also in the pathway commons interactome map, for the 276136 interactions between pairs of these genes defined by this interactome map. Genes are similarly clustered along the margins of the plot according to p-value. (e) Shows a zoomed-in view of the top-left of (c). (d) and (f) are as (c) and (e), but based on null p-values randomly sampled from a uniform (0,1) distribution, to demonstrate the structure present from the pathway commons interactome map, without the influence of the DNA methylation network interaction measure. Pearson correlation coefficients comparing values in these adjacency matrices as plotted, are as follows: (a) vs. (b), 0.0011; (c) vs. (d), 0.26; (e) vs. (f), 0.38. (JPG) [file pone.0084573.s001.jpg]
