# Supplementary material for: A DNA Methylation Network Interaction Measure, and Detection of Network Oncomarkers
Source: PLoS One. 2014 Jan 6;9(1):e84573. doi: 10.1371/journal.pone.0084573 (PMC3882261; doi:10.1371/journal.pone.0084573)
Supplement: Table S1 — Mitochondrial module. (PDF) [file pone.0084573.s002.pdf]

(a)

| Gene/node | Degree | Chr | Gene info |
|---|---|---|---|
| MRPL24 | 7 | 1 | mitochondrial ribosomal protein L24 |
| UQCRFS1 | 3 | 19 | ubiquinol-cytochrome c reductase, Rieske iron-sulfur polypeptide 1 |
| MRPL15 | 3 | 8 | mitochondrial ribosomal protein L15 |
| DNAJA3 | 2 | 16 | DnaJ (Hsp40) homolog, subfamily A, member 3 |
| DLD | 1 | 7 | dihydrolipoamide dehydrogenase |
| ETFA | 1 | 15 | electron-transfer-flavoprotein, alpha polypeptide |
| ISCA1 | 1 | 9 | iron-sulfur cluster assembly 1 homolog (S. cerevisiae) |
| MRPL44 | 1 | 2 | mitochondrial ribosomal protein L44 |
| MRPS23 | 1 | 17 | mitochondrial ribosomal protein S23 |
| MRPS5 | 1 | 2 | mitochondrial ribosomal protein S5 |
| VDAC3 | 1 | 8 | voltage-dependent anion channel 3 |
| MRPS35 | 1 | 12 | mitochondrial ribosomal protein S35 |
| ST6GALNAC6 | 1 | 9 | ST6 (alpha-N-acetyl-neuraminyl-2,3-beta-galactosyl-1,3)-N-acetylgalactosaminide alpha-2,6-sialyltransferase 6 |

(b)

| Gene set | OR (95% C.I.) | $q$-val |
|---|---|---|
| MIPS_55S_RIBOSOME_MITOCHONDRIAL | 190 (51-690) | 1.8e-07 |
| MOOTHA_HUMAN_MITODB_6_2002 | 53 (15-210) | 2.6e-06 |
| MOOTHA_MITOCHONDRIA | 51 (15-200) | 2.6e-06 |
| MIPS_28S_RIBOSOMAL_SUBUNIT_MITOCHONDRIAL | 150 (24-670) | 0.0064 |
| MITOCHONDRIAL_PART | 50 (11-180) | 0.0065 |
| MITOCHONDRION | 29 (7.3-100) | 0.0065 |
| MIPS_39S_RIBOSOMAL_SUBUNIT_MITOCHONDRIAL | 120 (20-530) | 0.0065 |
| MITOCHONDRIAL_MATRIX | 98 (16-410) | 0.0091 |
| MITOCHONDRIAL_LUMEN | 98 (16-410) | 0.0091 |
| STEIN_ESRRA_TARGETS_UP | 24 (6.1-83) | 0.011 |
| CHIANG_LIVER_CANCER_SUBCLASS_UNANNOTATED_DN | 34 (7.5-120) | 0.015 |
| MOOTHA_PGC | 21 (5.2-72) | 0.018 |
| STEIN_ESRRA_TARGETS | 17 (4.2-58) | 0.043 |

(a) Gene/node details, and (b) significantly enriched gene sets, for the mitochondrial module found as significant in the LUAD data set. $Q$-values in (b) indicate significance of enrichment in the corresponding gene set by the genes in this module, calculated according to a one-sided Fisher's exact test. Further details about these gene sets can be found from the website of the Broad Institute Molecular Signatures Database (*http://www.broadinstitute.org*).
