# Supplementary material for: A DNA Methylation Network Interaction Measure, and Detection of Network Oncomarkers
Source: PLoS One. 2014 Jan 6;9(1):e84573. doi: 10.1371/journal.pone.0084573 (PMC3882261; doi:10.1371/journal.pone.0084573)
Supplement: Table S2 — MAP-kinase module. (PDF) [file pone.0084573.s003.pdf]

(a)

Gene/node	Degree	Chr	Gene info
MAPK14	16	6	mitogen-activated protein kinase 14
SRC	2	20	v-src sarcoma (Schmidt-Ruppin A-2) viral oncogene homolog (avian)
CCDC14	1	3	coiled-coil domain containing 14
CCDC97	1	19	coiled-coil domain containing 97
DUSP16	1	12	dual specificity phosphatase 16
DUSP22	1	6	dual specificity phosphatase 22
DUSP6	1	12	dual specificity phosphatase 6
EEF2K	1	16	eukaryotic elongation factor-2 kinase
FGR	1	1	Gardner-Rasheed feline sarcoma viral (v-fgr) oncogene homolog
GMFB	1	14	glia maturation factor, beta
KLK3	1	19	kallikrein-related peptidase 3
MAP3K3	1	17	mitogen-activated protein kinase kinase kinase 3
MAP4K3	1	2	mitogen-activated protein kinase kinase kinase kinase 3
MLL3	1	7	myeloid/lymphoid or mixed-lineage leukemia 3
NFATC2	1	20	nuclear factor of activated T-cells, cytoplasmic, calcineurin-dependent 2
P2RY1	1	3	purinergic receptor P2Y, G-protein coupled, 1
PI4K2B	1	4	phosphatidylinositol 4-kinase type 2 beta
STK39	1	2	serine threonine kinase 39

(b)

Gene set	OR (95% C.I.)	q-val
PID_P38ALPHABETAPATHWAY	140 (35-450)	2.1e-05
PROTEIN_KINASE_CASCADE	25 (8-71)	0.00075
MAPKKK_CASCADE_GO_0000165	42 (11-130)	0.0016
INACTIVATION_OF_MAPK_ACTIVITY	190 (30-860)	0.0035
NEGATIVE_REGULATION_OF_MAP_KINASE_ACTIVITY	150 (25-680)	0.0039
KEGG_MAPK_SIGNALING_PATHWAY	21 (6.3-60)	0.0039
INTRACELLULAR_SIGNALING_CASCADE	13 (4.4-37)	0.0043
REACTOME_ADP_SIGNALLING_THROUGH_P2RY1	120 (20-510)	0.0054
REACTOME_SIGNAL_AMPLIFICATION	90 (15-360)	0.011
NEGATIVE_REGULATION_OF_TRANSFERASE_ACTIVITY	68 (12-260)	0.021
CELLULAR_PROTEIN_METABOLIC_PROCESS	8.4 (2.9-24)	0.049
CELLULAR_MACROMOLECULE_METABOLIC_PROCESS	8.3 (2.9-24)	0.049

(a) Gene/node details, and (b) significantly enriched gene sets, for the MAP-kinase module found as significant in the LUSC data set. Q-values in (b) indicate significance of enrichment in the corresponding gene set by the genes in this module, calculated according to a one-sided Fisher's exact test. Further details about these gene sets can be found from the website of the Broad Institute Molecular Signatures Database (*http://www.broadinstitute.org*).
