# Supplementary material for: A DNA Methylation Network Interaction Measure, and Detection of Network Oncomarkers
Source: PLoS One. 2014 Jan 6;9(1):e84573. doi: 10.1371/journal.pone.0084573 (PMC3882261; doi:10.1371/journal.pone.0084573)
Supplement: Table S3 — Largest biologically significant module, BLCA. (PDF) [file pone.0084573.s004.pdf]

(a)

Gene/node	Degree	Chr	Gene info
BSG	8	19	basigin (Ok blood group)
ATXN10	1	22	ataxin 10
ELTD1	1	1	EGF, latrophilin and seven transmembrane domain containing 1
HGSNAT	1	8	heparan-alpha-glucosaminide N-acetyltransferase
NPC1	1	18	Niemann-Pick disease, type C1
PPIB	1	15	peptidylprolyl isomerase B (cyclophilin B)
SDC1	1	2	syndecan 1
SLC16A4	1	1	solute carrier family 16, member 4 (monocarboxylic acid transporter 5)
SLC3A2	1	11	solute carrier family 3 (activators of dibasic and neutral amino acid transport), member 2

(b)

Gene set	OR (95% C.I.)	q-val
PID_SYNDECAN_1_PATHWAY	140 (21-680)	0.04

(a) Gene/node details, and (b) significantly enriched gene sets, for the largest biologically significant module in the BLCA data set. Q-values in (b) indicate significance of enrichment in the corresponding gene set by the genes in this module, calculated according to a one-sided Fisher's exact test. Further details about these gene sets can be found from the website of the Broad Institute Molecular Signatures Database (*http://www.broadinstitute.org*).
