# Supplementary material for: A DNA Methylation Network Interaction Measure, and Detection of Network Oncomarkers
Source: PLoS One. 2014 Jan 6;9(1):e84573. doi: 10.1371/journal.pone.0084573 (PMC3882261; doi:10.1371/journal.pone.0084573)
Supplement: Table S4 — Largest biologically significant module, BRCA. (PDF) [file pone.0084573.s005.pdf]

(a)

Gene/node	Degree	Chr	Gene info
ALPP	3	2	alkaline phosphatase, placental
AZU1	2	19	azurocidin 1
MPO	2	17	myeloperoxidase
ARL8B	2	3	ADP-ribosylation factor-like 8B
ALPPL2	1	2	alkaline phosphatase, placental-like 2

(b)

Gene set	OR (95% C.I.)	q-val
MARTINELLI_IMMATURE_NEUTROPHIL_UP	2300 (140-4.5e+15)	0.014
KEGG_FOLATE_BIOSYNTHESIS	920 (65-8200)	0.032

(a) Gene/node details, and (b) significantly enriched gene sets, for the largest biologically significant module in the BRCA data set. Q-values in (b) indicate significance of enrichment in the corresponding gene set by the genes in this module, calculated according to a one-sided Fisher's exact test. Further details about these gene sets can be found from the website of the Broad Institute Molecular Signatures Database (*http://www.broadinstitute.org*).
