# Supplementary material for: A DNA Methylation Network Interaction Measure, and Detection of Network Oncomarkers
Source: PLoS One. 2014 Jan 6;9(1):e84573. doi: 10.1371/journal.pone.0084573 (PMC3882261; doi:10.1371/journal.pone.0084573)
Supplement: Table S5 — KIRC large subnetwork. (PDF) [file pone.0084573.s006.pdf]

(a)

Gene/node	Degree	Chr	Gene info
ZNF638	76	2	zinc finger protein 638
SF3B4	55	1	splicing factor 3b, subunit 4, 49kDa
CPSF1	50	8	cleavage and polyadenylation specific factor 1, 160kDa
PRPF19	41	11	PRP19/PSO4 pre-mRNA processing factor 19 homolog (S. cerevisiae)
KPNA1	37	3	karyopherin alpha 1 (importin alpha 5)
EPPK1	37	8	epiplakin 1
PRPF6	35	20	PRP6 pre-mRNA processing factor 6 homolog (S. cerevisiae)
DDX42	34	17	DEAD (Asp-Glu-Ala-Asp) box polypeptide 42
FASN	34	17	fatty acid synthase
HSPA9	31	5	heat shock 70kDa protein 9 (mortalin)
PSMC6	28	14	proteasome (prosome, macropain) 26S subunit, ATPase, 6
CDC27	25	17	cell division cycle 27
SMARCC1	24	3	SWI/SNF related, matrix associated, actin dependent regulator of chromatin, subfamily c, member 1
BRWD1	23	21	bromodomain and WD repeat domain containing 1
ILF2	23	1	interleukin enhancer binding factor 2
GTF3C2	23	2	general transcription factor IIIC, polypeptide 2, beta 110kDa
CSNK2A1	22	20	casein kinase 2, alpha 1 polypeptide
DDX19A	22	16	DEAD (Asp-Glu-Ala-Asp) box polypeptide 19A
MED16	22	19	mediator complex subunit 16
POLR2E	21	19	polymerase (RNA) II (DNA directed) polypeptide E, 25kDa
MYC	20	8	v-myc myelocytomatosis viral oncogene homolog (avian)
TPX2	17	20	TPX2, microtubule-associated, homolog (Xenopus laevis)
TMOD3	17	15	tropomodulin 3 (ubiquitous)
RAC2	17	22	ras-related C3 botulinum toxin substrate 2 (rho family, small GTP binding protein Rac2)
ACTL6A	16	3	actin-like 6A
CFL1	16	11	cofilin 1 (non-muscle)
BUB1	15	2	BUB1 mitotic checkpoint serine/threonine kinase
ARCN1	15	11	archain 1
SRP19	15	5	signal recognition particle 19kDa
PRPF8	15	17	PRP8 pre-mRNA processing factor 8 homolog (S. cerevisiae)
PIK3CA	13	3	phosphatidylinositol-4,5-bisphosphate 3-kinase, catalytic subunit alpha
ERCC3	13	2	excision repair cross-complementing rodent repair deficiency, complementation group 3
PAK1	12	11	p21 protein (Cdc42/Rac)-activated kinase 1
RPS21	12	20	ribosomal protein S21
ITGA2	12	5	integrin, alpha 2 (CD49B, alpha 2 subunit of VLA-2 receptor)
NF1	12	17	neurofibromin 1
PEA15	12	1	phosphoprotein enriched in astrocytes 15
PSMB5	12	14	proteasome (prosome, macropain) subunit, beta type, 5

(b)

Gene set	OR (95% C.I.)	q-val
PUJANA_CHEK2_PCC_NETWORK	3.4 (2.7-4.3)	1.4e-19
PUJANA_BRCA1_PCC_NETWORK	2.6 (2.1-3.1)	1.6e-18
NUCLEUS	2.5 (2-3)	3.8e-16
NUCLEAR_PART	3.1 (2.3-3.9)	3.6e-12
DIAZ_CHRONIC_MEYLOGENOUS_LEUKEMIA_UP	2.2 (1.8-2.7)	4.8e-12
NUCLEOBASENUCLEOSIDENUCLEOTIDE_AND_NUCLEIC_ACID_METABOLIC_PROCESS	2.2 (1.8-2.6)	4.1e-10
REACTOME_APOPTOSIS	5 (3.3-7.4)	5.6e-10
INTRACELLULAR_ORGANELLE_PART	2.2 (1.8-2.7)	1.1e-09
REACTOME_HIV_INFECTION	4.4 (3-6.3)	1.1e-09
ORGANELLE_PART	2.2 (1.8-2.7)	1.2e-09
REACTOME_METABOLISM_OF_RNA	4.3 (2.9-6.2)	1.5e-09
REACTOME_METABOLISM_OF_MRNA	4.7 (3.1-7.1)	2.2e-09
PILON_KLF1_TARGETS_DN	1.9 (1.6-2.3)	3.3e-09
KEGG_SPLICEOSOME	5.7 (3.5-9.2)	8.7e-09
PENG_GLUTAMINE_DEPRIVATION_DN	3.3 (2.3-4.5)	1.2e-08
REACTOME_AUTODEGRADATION_OF_CDH1_BY_CDH1_APC_C	9.1 (4.8-17)	1.8e-08
MACROMOLECULAR_COMPLEX	2.2 (1.7-2.7)	2.7e-08
REACTOME_APC_C_CDH1_MEDIATED_DEGRADATION_OF_CDC20_AND_OTHER APC_C_CDH1_TARGETED_PROTEINS_IN_LATE_MITOSIS_EARLY_G1	7.9 (4.3-14)	3.8e-08
MITSIADES_RESPONSE_TO_APLIDIN_DN	3.5 (2.4-5)	4.1e-08
BIOPOLYMER_METABOLIC_PROCESS	1.9 (1.5-2.2)	4.2e-08
DODD_NASOPHARYNGEAL_CARCINOMA_DN	2.1 (1.7-2.6)	4.2e-08
DACOSTA_UV_RESPONSE_VIA_ERCC3_DN	2.2 (1.7-2.7)	4.9e-08
RNA_METABOLIC_PROCESS	2.2 (1.7-2.7)	8.5e-08
REACTOME_REGULATION_OF_MITOTIC_CELL_CYCLE	6.4 (3.7-11)	9.1e-08
REACTOME_APC_C_CDC20_MEDIATED_DEGRADATION_OF_MITOTIC_PROTEINS	7.2 (4-13)	1.1e-07

(a) Gene/node details for the top 5% of the degree distribution, and (b) top 25 most significantly enriched gene sets, for the large subnetwork found as significant in the KIRC data set. Q-values in (b) indicate significance of enrichment in the corresponding gene set by the genes in this subnetwork, calculated according to a one-sided Fisher's exact test. Further details about these gene sets can be found from the website of the Broad Institute Molecular Signatures Database (*http://www.broadinstitute.org*).
