# Supplementary material for: A DNA Methylation Network Interaction Measure, and Detection of Network Oncomarkers
Source: PLoS One. 2014 Jan 6;9(1):e84573. doi: 10.1371/journal.pone.0084573 (PMC3882261; doi:10.1371/journal.pone.0084573)
Supplement: Table S6 — LUAD large subnetwork. (PDF) [file pone.0084573.s007.pdf]

(a)

Gene/node	Degree	Chr	Gene info
YWHAZ	16	8	tyrosine 3-monooxygenase/tryptophan 5-monooxygenase activation protein, zeta polypeptide
YWHAG	16	7	tyrosine 3-monooxygenase/tryptophan 5-monooxygenase activation protein, gamma polypeptide
RAC1	14	7	ras-related C3 botulinum toxin substrate 1 (rho family, small GTP binding protein Rac1)
SRC	13	20	v-src sarcoma (Schmidt-Ruppin A-2) viral oncogene homolog (avian)
ELAVL1	12	19	ELAV (embryonic lethal, abnormal vision, Drosophila)-like 1 (Hu antigen R)
KHDRBS1	9	1	KH domain containing, RNA binding, signal transduction associated 1
AGK	8	7	acylglycerol kinase
RPLP0	8	12	ribosomal protein, large, P0
DDX19B	8	16	DEAD (Asp-Glu-Ala-Asp) box polypeptide 19B
INTS3	8	1	integrator complex subunit 3
ARID5B	7	10	AT rich interactive domain 5B (MRF1-like)
CYBA	7	16	cytochrome b-245, alpha polypeptide
BZW1	7	2	basic leucine zipper and W2 domains 1
MLL2	7	12	myeloid/lymphoid or mixed-lineage leukemia 2
SNX2	7	5	sorting nexin 2

(b)

Gene set	OR (95% C.I.)	q-val	
YWHAZ	16	8	tyrosine 3-monooxygenase/tryptophan 5-monooxygenase activation protein, zeta polypeptide
YWHAG	16	7	tyrosine 3-monooxygenase/tryptophan 5-monooxygenase activation protein, gamma polypeptide
RAC1	14	7	ras-related C3 botulinum toxin substrate 1 (rho family, small GTP binding protein Rac1)
SRC	13	20	v-src sarcoma (Schmidt-Ruppin A-2) viral oncogene homolog (avian)
ELAVL1	12	19	ELAV (embryonic lethal, abnormal vision, Drosophila)-like 1 (Hu antigen R)
KHDRBS1	9	1	KH domain containing, RNA binding, signal transduction associated 1
AGK	8	7	acylglycerol kinase
RPLP0	8	12	ribosomal protein, large, P0
DDX19B	8	16	DEAD (Asp-Glu-Ala-Asp) box polypeptide 19B
INTS3	8	1	integrator complex subunit 3
ARID5B	7	10	AT rich interactive domain 5B (MRF1-like)
CYBA	7	16	cytochrome b-245, alpha polypeptide
BZW1	7	2	basic leucine zipper and W2 domains 1
MLL2	7	12	myeloid/lymphoid or mixed-lineage leukemia 2
SNX2	7	5	sorting nexin 2

(a) Gene/node details for the top 5% of the degree distribution, and (b) top 25 most significantly enriched gene sets, for the large subnetwork found as significant in the LUAD data set. Q-values in (b) indicate significance of enrichment in the corresponding gene set by the genes in this subnetwork, calculated according to a one-sided Fisher's exact test.
